# Ovarian Development of Female-Female Pairs in the Termite, *Reticulitermes speratus*

**DOI:** 10.1673/031.010.19401

**Published:** 2010-11-08

**Authors:** Kyoko Ishitani, Kiyoto Maekawa

**Affiliations:** Graduate School of Science and Engineering, University of Toyama, Toyama 930–8555, Japan

**Keywords:** queen, parthenogenesis, juvenile hormone, vitellogenin, ovary, gene expression

## Abstract

In the rhinotermitid termite *Reticulitermes speratus* (Kolbe) (Isoptera: Rhinotermitidae), facultative parthenogenesis is known to occur occasionally and females cooperate with other females to found the colony. To elucidate the ovarian development in these two females, incipient female-female colonies were established under laboratory conditions, and the process of colony development was observed at 0.5, 1.5, 2.5, 3.5, and 7.5 months (stages I–V, respectively) after colony foundation. Ovarian development, vitellogenin gene expression, and juvenile hormone (JH) titers were examined. A precise reproductive cycle in both females was observed, in which the oviposition rate was relatively higher during stages I and II, decreased during stages III and IV, and then increased again at stage V. JH III titer and vitellogenin gene expression changed in parallel throughout the reproductive cycle of these queens. Ovarian maturation and vitellogenesis were similar in both females in a female-female colony at all stages examined, suggesting that no conflicts existed for two females in terms of oviposition.

## Introduction

Termite colonies are basically established by monogamous adult pairs in the swarming season (Nutting 1969). However, parthenogenestic female adults have also been observed in 8 species belonging to 4 families (Termopsidae: *Zootermopsis angusticollis, Z. nevadensis*, Kalotermitidae: *Kalotermes flavicollis*, Rhinotermitidae: *Reticulitermes virginicus, R. speratus, R. hesperus, Velocitermes* sp., Termitidae: *Cubitermes ugandensis*) and is suspected in 2 species (*K. beesoni* ana *R. lucifugus*) (Nutting 1969; Matsuura and Kobayashi 2007).

Within these species, *R. speratus*, which is one of the most common termite species in Japan, has been examined in detail with regard to the mode of parthenogenesis. Matsuura and Nishida (2001) found that females that failed to pair with males could reproduce by parthenogenesis. Although these facultatively parthenogenetic eggs had longer hatching periods than sexually produced eggs, both asexual and sexual eggs had similar hatching rates (Matsuura and Kobayashi 2007). When female-female pairs were established, two females produced equal numbers of offspring by 100 days after colony foundation (Matsuura et al. 2002). Moreover, the number of first-brood offspring in a female-female colony 50 days after colony foundation were significantly less than those after 400 days, but after 400 days in a female-male colony the number of offspring were not significantly different from those at 50 days (Matsuura et al. 2004). These results suggest a lower survival rate of parthenogenetically produced individuals than sexually produced ones (Matsuura et al. 2004). These previous studies showed that there were no significant differences in queen survivorship and number of offspring between nestmate pairs and nonnestmate pairs in both female-female and female-male colonies.

Development of the incipient female-male colony has been studied in some termite species, and the reproductive cycles of queens were shown in them (reviewed in Nutting 1969). In *R. speratus*, after the establishment of female-male pairs under laboratory conditions, the reproductive cycle in queens has been observed (Maekawa et al. 2010). It was suggested that queens probably stopped laying eggs by 2.5 months after colony foundation, and resumed oviposition at some point prior to 7.5 months when the colony contained sufficient workers. Throughout such a reproductive cycle in *R. speratus* queens, ovarian development and vitellogenin gene expression were shown to change in parallel. Moreover, juvenile hormone (JH) III titer changes were also shown to be correlated with transcription of the vitellogenin gene and vitellogenesis in ovaries at least after the imaginal molt to the repression of egg production at 2.5 months after colony foundation. However, it is still unclear whether these reproductive cycles of queens are observed in female-female colonies. There is a possibility that different reproductive cycles of females in two-queen colonies may cause the reduction in the numbers of offspring after colony foundation. It is also possible that 2 females found a colony cooperatively at the beginning, but then reproductive conflict between the 2 females occurs from around 100 days after colony foundation. For example, one queen resumes oviposition and the other stops laying eggs and performs altruistic helping behaviors. In order to understand these issues, the ovarian development of each queen in a female-female colony should be examined in detail.

In this study, ovarian development of each female of the rhinotermitid *Reticulitermes speratus* (Kolbe) was monitored and JH titer was measured after the establishment of a female-female colony examine the reproductive cycles of each queen and determine if reproductive conflict between 2 queens occurs. The development of incipient colonies was determined by recording colony composition at 0.5, 1.5, 2.5, 3.5, and 7.5 months (stages I–V, respectively) after colony foundation. Queens were sampled at each stage to quantify ovarian development, expression of the vitellogenin gene, and JH titer. These data were compared to those of queens in female-male colonies reported in the previous study (Maekawa et al. 2010).

## Materials and methods

### Termites

Four mature termite colonies were collected from Kureha Hill in Toyama Prefecture, Japan, in May 2008. Pieces of logs were brought back to the laboratory and kept in plastic cases in constant darkness. Sixth-instar nymphs (N6; Takematsu 1992; Maekawa et al. 2008) with swollen wing buds, just prior to molt into alates were picked from each colony.

### Female-female colony foundation

After the emergence of alates, the sexes of termite individuals were discriminated according to the morphology of their abdominal tergites (Weesner 1969). Dealated adults were chosen randomly and femalefemale pairs were made. All females used were picked from the same colony (total 556 pairs). Each pair was placed in a 20 ml glass vial with c. 8 g of mixed sawdust food (Mitani, www.e-musi.co.jp) and kept at 25°° C in constant darkness. Colonies were then sampled after 0.5, 1.5, 2.5, 3.5, and 7.5 months (stage I–V, respectively). For measurements of each colony size (numbers of eggs, larvae, workers, soldiers, and nymphs) and histological observations of the ovaries (see below), samples were fixed in formaldehyde/ethanol/acetic acid (6:16:1, v/v/v) for at least 24 h and stored in 70% ethanol. For RNA and JH extraction (see below), samples were immersed immediately in liquid nitrogen and stored at -80°° C until use. Female-male colonies were constructed using 4 colonies under the condition as one female from one colony and one male from another (total 543 pairs; Maekawa et al. 2010). The colony size and JH titer level data were obtained to compare with those of female-female colonies.

### Histological observations

To describe the histological characteristics of ovaries in each queen, paraffin-embedded sections were made and stained with hematoxylin and eosin. Abdomens preserved in 70% ethanol were dehydrated in increasing concentrations of ethanol, then transfer to xylene, and finally embedded in paraffin. Serial parasagittal sections (10 μ?m thick) were cut using an MRS80–074 microtome (Ikemoto, www.ikemoto.co.jp) and stained with hematoxylin and eosin. The numbers of abdomens sectioned were 12 (= 6 colonies, stage I), 8 (= 4 colonies, stage II), 6 (= 3 colonies, stage III), 10 (= 5 colonies, stage IV), and 12 (= 6 colonies, stage V). Tissues on slides were observed using BX-40 (Olympus, www.olympus.com) and BZ-8100 (Keyence, www.keyence.com) microscopes. The numbers of vitellogenic oocytes were counted in each individual, and these numbers were used to evaluate the degree of ovarian maturation.

### cDNA preparation and real-time quantitative PCR

Total RNA was extracted from individual termites stored at -80°° C using a FastPure RNA kit (Takara Bio, www.takara-bio.com). Six queens from three colonies were used at each stage. Three different alate individuals were also examined. For single strand cDNA synthesis, DNase-treated mRNA was reverse transcribed using a SuperScript First-Strand Synthesis System for RT-PCR (Invitrogen, www.invitrogen.com) following the manufacturer's instructions. The relative quantifications of transcripts were performed using SYBR Green I reagent and a MiniOpticon Real-Time System (Bio-Rad, www.bio-rad.com). *Beta-actin* was used as the endogenous control gene, because this gene was evaluated as the most reliable reference gene in *R. flavipes* (Scharf et al. 2005; Zhou et al. 2006). Primers for the target *vitellogenin I* (GenBank accession number: AB520715) and the control *beta-actin* (AB520714) genes were reported by Maekawa et al. (2010).

### JH extraction

Experimental details were reported by Maekawa et al. (2010). Briefly, five individuals stored at -80°°C were homogenized in 1 ml methanol/isooctane (1:1, v/v) and allowed to stand at room temperature for 30 min. Eight replicate samples (= 40 individuals) were prepared per each developmental stage. After centrifugation at 13,900 g for 5 min, the supernatant was collected and mixed with 10 µµg of fenoxycarb (Wako, www.wako-chem.co.jp) as an internal standard. The mixture was vortexed and allowed to stand at room temperature for 30 min, before centrifugation at 5,600 g for 15 min. The upper isooctane phase was transferred into a new glass vial, and the methanol phase was vortexed and centrifuged at 7,700 g for 30 min, and then combined with the isooctane phase. The resulting mixture was stored at -80°° C before vacuum drying in a CC-181 centrifugal concentrator (Tomy, www.tomytech.com). Dried pellets were dissolved in 20 μ?l of methanol.

### Liquid chromatography-mass spectrometry (LC-MS)

From each 20 μ?l concentrated sample, 5 μ?l was separated using a 150 ×× 2 mm^2^ C18 reverse-phased column (YMC-Pack Pro C18.5 μ?m, YMC Co. Ltd., www.ymc.co.jp) protected by a guard column (YMC-Pack Pro, sphere ODS, YMC Co. Ltd.) with a gradient elution of water/methanol (0–15 min 80–100% methanol, 15–20 min 100% methanol) at a flow rate of 0.2 ml/min, utilizing an Agilent 1100 HPLC system with an autosampler (Agilent Technologies, www.agilent.com). Mass spectral analysis was performed by electrospray ionization (ESI) in the positive mode using a microTOF-HS (Bruker Daltonics GmbH, www.Bruker.com) under the following conditions: the electrospray capillary was set at 4.5 kV and the dry temperature was 200°° C. The nitrogen pressure for the nebulizer was 1.6 bar and the drying gas nitrogen flow-rate was 9 L/min. Quantification of JH III and fenoxycarb was performed by monitoring the [M+H]+ and [M+Na]+ ions. A calibration curve for JH III (Sigma-Aldrich, www.sigmaaldrich.com) was plotted using the same internal standard concentration as fenoxycarb in each sample. The JH III titer from each sample was then calculated after analysis of the chromatogram data using QuantAnalysis software (Bruker Daltonics). Results were expressed as ng/mg wet weight.

### Statistical tests

For the comparisons of colony development and JH titer of queens in each developmental stage between female-female and female-male colonies, a statistical post-hoc test was performed using the Mann-Whitney U test. For the comparisons of oocyte numbers per queen in each colony, t test was conducted. A statistical comparison of gene expression levels among stages was performed using a non-parametric Steel-Dwass test. Each test was performed using the statistical software Mac Statistical Analysis ver. 1.5 (Esumi, www.esumi.co.jp). p values less than 0.05 were considered significant.

**Figure 1.**
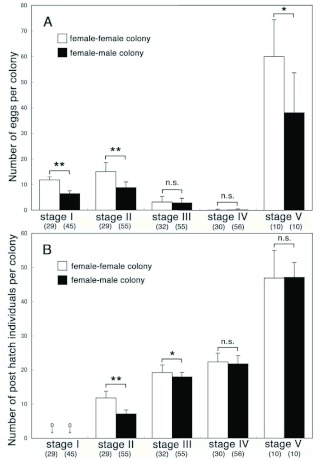
Mean number (mean ±± S.D. values) of eggs (A) and post-hatching individuals (including larvae, workers, presoldiers, soldiers, and nymphs) per colony (B) at 0.5, 1.5, 2.5, 3.5, and 7.5 months (stages l–V, respectively) after colony foundation. The numbers of *Reticulitermes speratus* colonies examined are indicated in parentheses. Data of female-male colonies were obtained from the previous report (Maekawa et al. 2010). Asterisks denote significant differences between female-female and femalemale colonies at each stage (Mann-Whitney U test, **p<0.05, **** p<0.0l). High quality figures are available online.

## Results

### Number of eggs and offspring per colony

Figure 1 shows the numbers of eggs (A) and post-hatching individuals (larvae, workers, presoldiers, soldiers, and nymphs) (B) per colony for each stage. Data of female-male colonies were obtained from Maekawa et al. (2010). The numbers of eggs in each femalefemale colony were significantly larger than those in female-male colonies at stages I and II (p < 0.0001). The numbers of eggs in both colonies decreased at stages III and IV, and no significant differences were found (stage III: p = 0.68; stage IV: p = 0.24). At stage V, many eggs were observed again in both colonies, and the numbers in female-female colonies were significantly larger than those in femalemale colonies (p = 0.01). Post-hatching individuals were observed after stage II in both female-female and female-male colonies. The numbers of them per female-female colony were significantly larger than those per female-male colony at stages II (p < 0.0001) and III (p = 0.01). Only a single presoldier, which molted into a soldier, was observed in 2 female-female colonies at stage III (2/32 colonies: 6%), and one soldier was observed at stage IV in all female-female colonies examined (30/30 colonies: 100%). At stage IV, 19 female-female colonies (63%) contained nymphs (1-3 individuals per colony), which were never observed in female-male colonies (Maekawa et al. 2010). During stage V, 2 soldiers were observed in 5 of 10 female-female colonies, and the remaining 5 colonies had only one soldier. Nymphs were observed in 3 of 10 femalefemale colonies at stage V. The numbers of post-hatching individuals per female-female colony were not significantly different from those per female-male colony during stages IV (p = 0.26) and V (p = 0.79).

### Ovarian development

**Figure 2.**
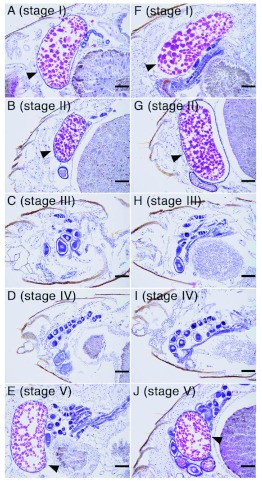
Parasagittal sections of *Reticulitermes speratus* abdomens at each developmental stage (AF: stage I, BG: stage II, CH: stage III, Dl: stage IV, EJ: stage V). Sections of two females from the same female-female colony are shown at each stage. Vitellogenic basal oocytes were observed in the ovarioles of both queens at stages I, II, and V (stained in red marked by arrowheds). Each bar indicates 100 μ?m. High quality figures are available online.

Vitellogenic basal oocytes were observed in the ovaries of queens at stages I, II, and V (Figures. 2AF, BG, and EJ), when many eggs were found in their colonies (Figure 1A). At stages III and IV, vitellogenic oocytes were observed rarely in the ovaries of queens (Figures 2CH and 2DI). Ovarian developmental state was similar to each other in each female-female pair at all stages. Indeed, the mean number of vitellogenic oocytes (mean ±± S.D.) in one queen (more oocytes were observed) and the other queen (less oocytes were observed) were as follows; 3.3 ±± 1.0 and 2.0 ±± 1.1 (stage I, n = 6 colonies), 3.5 ±± 0.6 and 2.3 ±± 0.5 (stage II, n = 4), 1.0 ±± 1.7 and 0.3 ±± 0.6 (stage III, n = 3), 0.2 ±± 0.4 and 0.0 ±± 0.0 (stage IV, n = 5), 4.0 ±± 1.7 and 3.3 ±± 1.2 (stage V, n = 6). Numbers of oocytes per one queen in a colony were not significantly different from those per the other queen (t test; stage I: p = 0.17; stage II: p = 0.08; stage III: p = 0.42; stage IV: p = 0.37; stage V: p = 1.00).

### Vitellogenin gene expression

**Figure 3.**
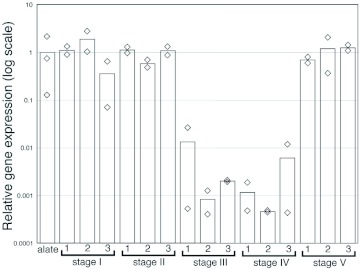
Expression levels of the *Reticuliermes speratus* vitellogenin gene analyzed by real-time quantitative PCR. The relative expression levels were calibrated using the mean expression level of alates as 1.0. Diamonds and bars indicate the level of each female and the mean (n = 2 or 3), respectively. Three female-female colonies (= 6 females) were examined at each stage. High quality figures are available online.

Figure 3 shows the relative expression levels of the vitellogenin gene analyzed by real-time quantitative PCR. The vitellogenin gene showed higher expression levels in alates and queens at stages I, II, and V, and lower expression levels in queens at stages III and IV. Expression levels were generally similar in each female in a female-female colony. Although more than 10-fold different levels were observed in each female in colony 1 (stage III) and colony 3 (stage IV), the expression levels were quite low in all these females (10^2–3^-fold lower than the levels observed in alates). When the Steel-Dwass test was performed among alates (n = 3) and other stages (n = 6) using pooled data, expression levels in queens at stages III and IV were significantly different from those at stages I, II and V (\*T*\ = 2.8823; 0.01 < p < 0.05).

### JH titer quantification

**Figure 4.**
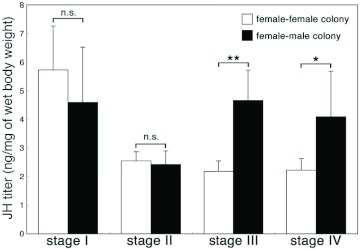
Juvenile hormone III titers of *Reticulitermes speratus* individuals at each developmental stage (mean ±± S. D. values). Each sample was pooled from 5 individuals, with 8 replicates per stage (= 40 individuals). Data of female-male colonies were obtained from the previous report (Maekawa et al. 2010). Asterisks denote significant differences between female-female and female-male colonies at each stage (Mann-Whitney U test, **p<0.05, **** p<0.0l). High quality figures are available online.

The LC-MS results are shown in Figure 4. The highest JH levels were observed in queens at stage I. Then, a decrease in JH titer in queens at stage II was observed, and relatively low JH titers were maintained during stages III and IV. JH titer levels observed in queens in female-female colonies at stages III and IV were significantly lower than those in queens in female-male colonies (stage III: p = 0.0008; stage IV: p = 0.0087), although no significant differences were found at stage I (p = 0.21) and II (p = 0.34). Unfortunately, JH titer of queens at stage V could not be determined because of the shortage of samples.

## Discussion

### Reproductive cycles in queens of femalefemale colony

The reproductive cycle in queens of *R. speratus* were observed previously in femalemale colonies under laboratory conditions (Maekawa et al. 2010). The queens stopped laying eggs between 1.5 and 2.5 months after colony foundation and then resumed it sometime prior to 7.5 months after colony foundation. Similar behavioral changes in queens were observed in female-female incipient colonies under laboratory conditions in the present study. At stages III and IV, vitellogenic oocytes were rarely observed in the ovaries of queens, the queens might have ceased laying eggs. At stage V, there were many eggs in the colonies and vitellogenic oocytes were observed again in the queens' ovaries, suggesting that the queens probably resumed oviposition earlier than stage V. Real-time quantitative PCR analyses in this study also showed that ovarian development and vitellogenin gene expression changed in parallel throughout the reproductive cycle of both queens in a female-female colony.

### Ovarian developments of each queen in female-female colony

The numbers of eggs per female-female colony were significantly larger than those per female-male colony at stages I, II, and V, when queens actively laid eggs. The ovarian developmental state and vitellogenin gene expression of one female were quite similar to those of the other female in all female-female colonies examined. These results strongly suggested that there were rarely reproductive conflicts at the time of oviposition between two females. Based on mitochondrial DNA RFLP analysis, Matsuura et al. (2002) showed that the numbers of offspring produced by each female did not differ significantly from each other at 100 days after foundation of a female-female colony. The present results were not inconsistent with these previous genetic data. The numbers of post-hatching individuals per female-female colony were similar to those per female-male colony after stage III. This was probably because of a lower survival rate of parthenogenetic offspring than sexually produced offspring, as suggested by Matsuura et al. (2004). It is also possible that a queen may interfere with offspring of a partner queen in a female-female colony, causing increased mortality. In contrast, the male may contribute directly to caring for the offspring (see below), ensuring a higher survival rate.

### Caste differentiation in female-female colony

Nymphs were observed only in female-female colonies at stages IV and V, suggesting that parthenogenetic eggs produced by adult females were influenced by genetic factors concerned with genetic caste determination between nymphs and workers found in this species (Hayashi et al. 2007). However, the relationships between the remaining castes in a colony and their genotypes are still unclear. Soldiers and nymphs were observed at stages IV and V, although they were not a workforce in the colony. Nymphs could molt into secondary nymphoid reproductives (Miyata et al. 2004; Maekawa et al. 2008), and the nymphoids asexually produced by the primary queen were involved in the main reproductive-force in natural colonies (Matsuura et al. 2009). If the queens would like to produce nymphoids, conflicts affecting nymphal development might exist between 2 queens in female-female colonies. To understand the relationship between reproductive conflict between two females and caste differentiation of their offspring, further analyses of the genotypes of individuals in a female-female colony after stage III are needed.

### JH titer levels of queens in female-female and female-male colonies

The JH III titer was correlated strongly with vitellogenin expression in addition to the actual vitellogenesis in the ovaries of both queens in female-female colonies. The increase in JH titer was correlated positively with the high levels of vitellogenin expression in queens at stages I and II. Then, the decrease in JH titer in queens at stage II preceded the decline in vitellogenesis at stages III and IV. These changes were also observed in queens of female-male colonies of *R. speratus* (Maekawa et al. 2010). Consequently, ovarian development and the reproductive cycle of queens in female-female and female-male colonies were suggested to be regulated by JH. However, JH levels at stages III and IV of queens in female-male colonies were relatively high (about 4 ng/mg of wet body weight), compared with those of queens in female-female colonies at the stages shown in the present study (about 2 ng/mg; Figure 4). Such differences in JH titer level could have been made possible by the existence of a male in the sexual colonies. For example, the ovarian maturation of female primary reproductives of *Z. angusticollis* was promoted by contact with a reproductive male within 60 days after colony initiation (Brent and Traniello, 2001a). Moreover, JH production rates significantly decreased in female reproductives paired with a male from 5 to 30 days. On the other hand, JH production rates in females paired with a female did not change significantly during 0–15 days (Brent et al. 2007). Consequently, it was suggested that the JH titer of female reproductives was influenced by the social stimuli, probably released by a male. It is also possible that males can also effect the females by contributing nutrients via trophallaxis (e.g. nitrogen-rich substances; Shellman-Reeve, 1990) or saving the females energy by helping to care for offspring which paired female might not contribute.

An alternative explanation was that the numbers of worker per queen in a femalemale colony are about 2-fold larger than those in a female-female colony at stages III and IV (Figure 1B), thus queens in female-male colonies could receive more nutrients than queens in female-female colonies. Indeed, in *Z. angusticollis*, both primary and secondary queens nesting with workers (in this case, 3rd and 4th-instar larvae) had higher fecundities than those lacking workers (Brent and Traniello, 2001b). JH titers were probably strongly influenced by the nutritional states of queens. It is also very interesting to know whether JH titer response is continuous as worker number increases or if it is dependent on threshold numbers being present. To understand these issues, JH titers of queens in female-female and female-male colonies of different sizes should be examined.

## Conclusion

The reproductive cycle in queens of *R. speratus* was observed in female-female colonies under laboratory conditions, as used for female-male colonies in the previous study. JH titer and vitellogenin gene expression changed in parallel throughout the reproductive cycle of both queens in femalefemale colonies. Ovarian development and vitellogenesis of each queen in female-female colonies were similar to each other, suggesting that no conflicts existed for two females in terms of oviposition.

## References

[bibr01] Brent CS, Traniello JFA (2001a). Influence of sex-specific stimuli on ovarian maturation in primary and secondary reproductives of the dampwood termite *Zootermopsis angusticollis*.. *Physiological Entomology*.

[bibr02] Brent CS, Traniello JFA (2001b). Social influence of larvae on ovarian maturation in primary and secondary reproductives of the dampwood termite *Zootermopsis angusticollis*.. *Physiological Entomology*.

[bibr03] Brent CS, Schal C, Vargo EL (2007). Endocrine effects of social stimuli on maturing queens of the dampwood termite *Zootermopsis angusticollis*.. *Physiological Entomology*.

[bibr04] Hayashi Y, Lo N, Miyata H, Kitade O (2007). Sex-linked genetic influence on caste determination in a termite.. *Science*.

[bibr05] Nutting WL, Krishna K, Weesner FM (1969). Flight and colony foundation.. *Biology of Termites*.

[bibr06] Maekawa K, Mizuno S, Koshikawa S, Miura T (2008). Compound eye development during caste differentiation of the termite *Reticulitermes speratus* (Isoptera: Rhinotermitidae).. *Zoological Science*.

[bibr07] Maekawa K, Ishitani K, Gotoh H, Cornette R, Miura T (2010). Juvenile Hormone titre and vitellogenin gene expression related to ovarian development in primary reproductives, as compared to nymphs and nymphoid reproductives of the termite *Reticulitermes speratus*.. *Physiological Entomology*.

[bibr08] Matsuura K, Kobayashi N (2007). Size, hatching rate, and hatching period of sexually and asexually produced eggs in the facultatively parthenogenetic termite *Reticulitermes speratus* (Isoptera: Rhinotermitidae).. *Applied Entomology and Zoology*.

[bibr09] Matsuura K, Nishida T (2001). Comparison of colony foundation success between sexual pairs and female asexual units in the termite *Reticulitermes speratus* (Isoptera: Rhinotermitidae).. *Population Ecology*.

[bibr10] Matsuura K, Fujimoto M, Goka K, Nishida T (2002). Cooperative colony foundation by termite female pairs: altruism for survivorship in incipient colonies.. *Animal Behaviour*.

[bibr11] Matsuura K, Fujimoto M, Goka K (2004). Sexual and asexual colony foundation and the mechanism of facultative parthenogenesis in the termite *Reticulitermes speratus* (Isoptera, Rhinotermitidae).. *Insectes Sociaux*.

[bibr12] Matsuura K, Vargo EL, Kawatsu K, Labadie PE, Nakano H, Yashiro T, Tsuji K (2009). Queen succession through asexual reproduction in termites.. *Science*.

[bibr13] Miyata H, Furuichi H, Kitade O (2004). Patterns of neotenic differentiation in a subterranean termite, *Reticulitermes speratus* (Isoptera: Rhinotermitidae).. *Entomological Science*.

[bibr14] Scharf ME, Scharf DW, Zhou XG, Pittendrigh BR, Bennett GW (2005). Gene expression profiles among immature and adult reproductive castes of the termite *Reticulitermes flavipes*.. *Insect Molecular Biology*.

[bibr15] Shellman-Reeve JS (1990). Dynamics of biparental care in the dampwood termite, *Zootermopsis nevadensis* (Hagen): response to nitrogen availability.. *Behavioral Ecology and Sociobiology*.

[bibr16] Takematsu Y (1992). Biometrical study on the development of the castes in *Reticulitermes speratus* (Isoptera, Rhinotermitidae).. *Japanese Journal of Entomology*.

[bibr17] Weesner FM, Krishna K, Weesner FM (1969). The reproductive system.. *Biology of Termites*.

[bibr18] Zhou X, Oi FM, Scharf ME (2006). Social exploitation of hexamerin: RNAi reveals a major caste-regulatory factor in termites.. *Proceedings of the National Academy of Sciences of the United States of America*.

